# Use of Vascular Shunt at the Time of Pancreatectomy with Venous Resection: A Systematic Review

**DOI:** 10.3390/cancers16132361

**Published:** 2024-06-27

**Authors:** Annarita Libia, Tiziana Marchese, Stefano D’Ugo, Prisco Piscitelli, Fabio Castellana, Maria Lisa Clodoveo, Roberta Zupo, Marcello Giuseppe Spampinato

**Affiliations:** 1 General Surgery Unit, Vito Fazzi Hospital, 73100 Lecce, Italy; 2 Department of Biological and Environmental Sciences and Biotechnologies, University of Salento, 73100 Lecce, Italy; 3 Local Health Authority, ASL LE, 73100 Lecce, Italy; 4 Department of Interdisciplinary Medicine, University of Bari “Aldo Moro”, Piazza Giulio Cesare 11, 70100 Bari, Italy

**Keywords:** pancreatic ductal carcinoma, vascular shunt, pancreatectomy, vascular resection, systematic review

## Abstract

**Simple Summary:**

This manuscript systematically reviewed the literature on the use of vascular shunts during advanced pancreatic surgery, analyzing intraoperative and postoperative outcomes, and enlightening excellent long-term patency, negligible additional operative time, and acceptable postoperative morbidity. The importance of the study was to underline feasibility of this technical artifice, which may help expert surgeons to achieve clear margins in borderline or locally advanced PDAC.

**Abstract:**

Background: The rising diffusion of vascular resections during complex pancreatectomy for malignancy, for both oncological and technical matters, brought with it the use of vascular shunts, either temporary or definitive, to prevent bowel congestion and liver ischemia. This study aimed to systematically review the literature on the technical feasibility of vascular shunts during advanced pancreatic surgery, analyzing intraoperative and postoperative outcomes. Methods: A systematic literature search was performed on PubMed, Scopus, Web of Science, and the Cochrane Library Central, according to PRISMA guidelines. Studies published before 2006 were excluded, considering the lack of a standardized definition of locally advanced pancreatic cancer. The main outcomes evaluated were the overall complication rate and shunt patency. Results: Among 789 papers retrieved from the database search, only five fulfilled the inclusion criteria and were included in the review, amounting to a total of 145 patients undergoing a shunt creation at the time of pancreatectomy. Pancreatic adenocarcinoma (PDAC) was found to be the most common diagnosis and pancreaticoduodenectomy was the main surgical procedure, accounting for 88% and 83% of the overall cohort, respectively. The distal splenorenal shunt was the most performed. Overall, 44 out of 145 patients (30%) experienced postoperative complications; the long-term patency of definitive shunts was 83% (110 out of 120 patients). Conclusions: An increasing number of patients with borderline resectable or locally advanced PDAC are becoming amenable to resection and shunt creation may facilitate vascular resection with clear margins, becoming a valid tool of modern pancreatic surgery.

## 1. Introduction

Pancreatic ductal carcinoma (PDAC) has a dismal prognosis, with a five-year survival rate of 5 to 8% [1]. Less than 20% of patients have resectable disease at the time of diagnosis, with five-year survival estimated at 20% after surgery [2,3]. However, in recent years, patients with initially unresectable diseases have been candidates for surgery as a result of advancements in multiagent chemotherapy and surgical techniques, with acceptable morbidity and mortality at high-volume referral centers [4]. Patients with borderline resectable or locally advanced PDAC require a more complex pancreatectomy, which usually involves vascular resections to achieve clear surgical margins (R0).

### 1.1. The Role of Surgical Shunts in the Era of Complex Pancreatic Surgery

Operative portosystemic shunts were first described for the treatment of gastroesophageal bleeding in end-stage liver disease complicated by extrahepatic portal hypertension [5]. With the introduction of TIPS and endoscopic banding, operative shunts were abandoned, but can still represent an option in case of neoplastic occlusion of the splenic-mesenteric-portal confluence (SMP confluence) during advanced pancreatic surgery.

### 1.2. The Mesocaval Shunt

**Figure 1:**
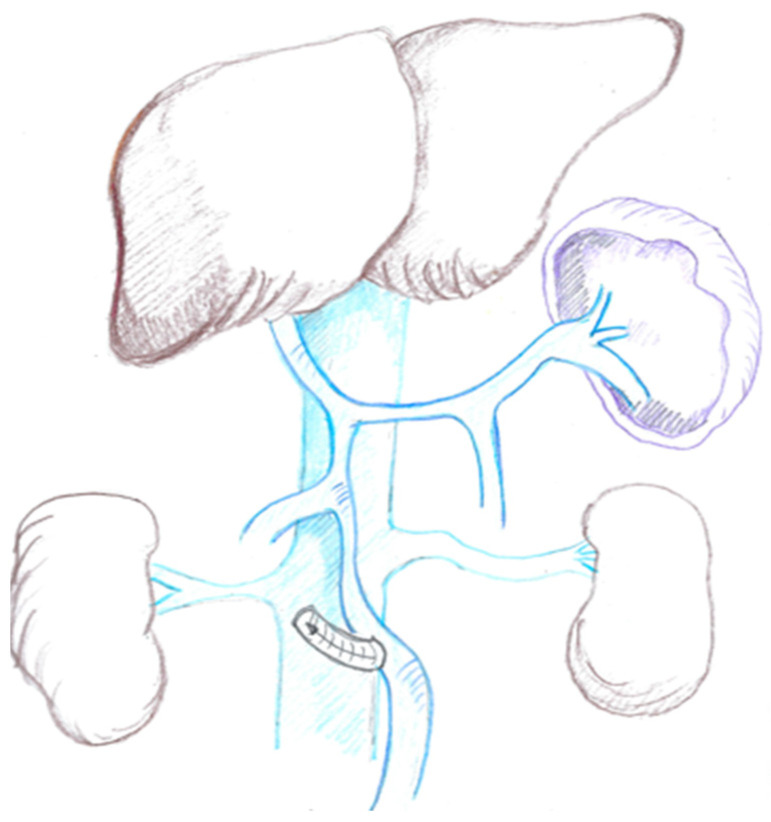
Mesocaval shunt (MCS).

In the setting of cavernous transformation of the portal vein (PV), dissection of SMP confluence in the presence of large venous collaterals might be life-threatening without first diverting mesenteric blood flow [6,7] towards systemic circulation through a mesocaval shunt (MCS) (Figure 1), which creates communication between inferior vena cava (IVC) and superior mesenteric vein (SMV) through an interposition graft, generally the left internal jugular vein (LIJV). Moreover, vascular resections imply prolonged PV and SMV clamping, which allow the specimen removal en bloc with the involved segment of SMP confluence but may result in bowel congestion.

### 1.3. The Mesoportal Shunt

**Figure 2:**
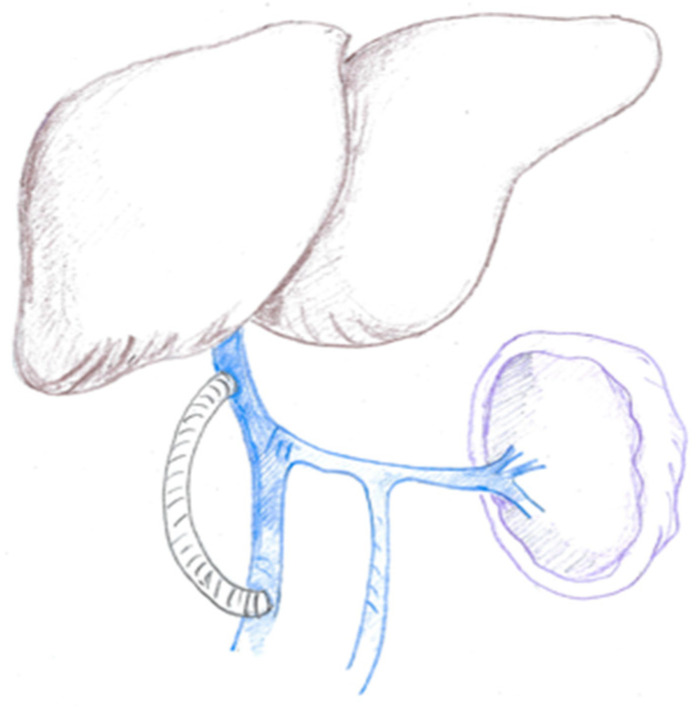
Mesoportal Shunt (MPS).

MCS can ensure midgut decompression but does not overcome liver ischemia due to portal clamping. In such cases, a mesoportal shunt (MPS) (Figure 2) can provide small bowel decompression maintaining liver perfusion and avoiding possible reperfusion injury, especially when preoperative liver function may have been altered by jaundice or neoadjuvant chemotherapy. In addition to mesenteric venous hypertension and the requirement of midgut decompression, vascular shunts may be useful in cases of tumor abutment to the posterior aspect of the superior mesenteric artery (SMA), since they enhance the exposure of the root of mesentery and arterial dissection. MCS is usually dismantled after tumor removal, restoring hepatopetal flow.

### 1.4. The Distal Spleno-Renal Shunt and the Underestimate Impact of Left-Sided Portal Hypertension

**Figure 3:**
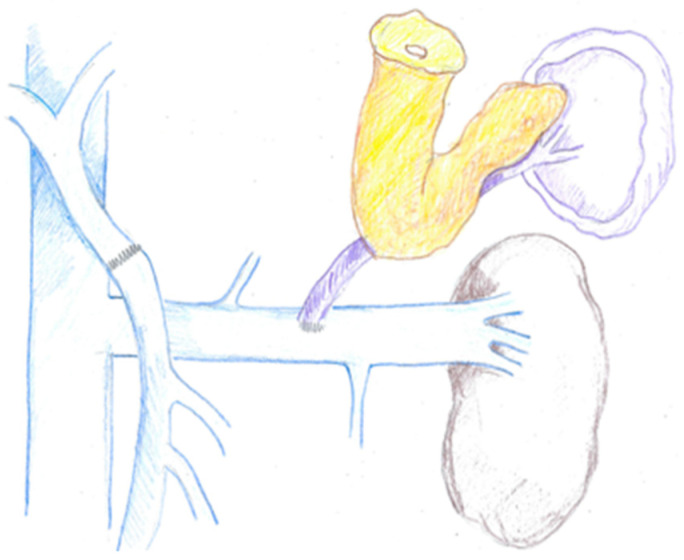
Distal spleno-renal shunt (DSRS).

Besides the mesocaval and mesoportal types, shunts also include the distal spleno-renal shunt (DSRS) (Figure 3), an end-to-side anastomosis between the splenic vein and left renal vein (LRV), first described by Warren in 1967 [7]. Splenic vein ligation may occur during pancreatic resections for different reasons, either oncological or technical. On one hand, it might be necessary to divide SV in case of neoplastic infiltration, on the other hand, splenic vein ligation may improve mobility of PV and SMV to perform a tension-free anastomosis without any interposition grafts; SV division may finally improve exposure of the surgical field in case of difficult SMA dissection. Nevertheless, splenic vein division may impair venous drainage from the spleen and stomach, especially when IMV enters SMV, thus predisposing patients to left-sided portal hypertension (LPH). Sinistral or left-sided portal hypertension is a peculiar type of portal hypertension where the splenic bloodstream is diverted through an alternative route, possibly entailing acute or chronic bleeding from varices (e.g., esophageal, gastric, jejunal), unexplained splenomegaly, and mild thrombocytopenia. Moreover, postoperative gastrointestinal bleeding secondary to LPH may be refractory to transfusions and may require delayed creation of a shunt. Several reports are described in the literature, where DSRS or MCS were surgically performed months or years after index surgery to treat refractory variceal bleeding [6].

In this context, where borderline or locally advanced PDAC are increasingly candidates for surgery, this systematic review aimed to ascertain the technical feasibility of operative shunts in advanced pancreatic surgery requiring vascular resection and reconstruction, analyzing intraoperative and postoperative outcomes.

## 2. Materials and Methods

### 2.1. Database Search

A systematic literature search was performed independently by two of the manuscript authors (AL, TM) on PubMed, Scopus, Web of Science, and Cochrane Library Central. For the PubMed database, the following combination was used: (“pancreatectomy” [MeSH Terms] OR “pancreaticoduodenectomy” [MeSH Terms] OR “pancreatic surgery”) AND (“shunt” [All Fields] OR “shunting” [All Fields] OR “shunts” [All Fields] OR “shunt*” [All Fields]). For the Web of Science database, the following combination was used: (shunt OR shunting OR shunts) AND (pancreatectomy OR pancreaticoduodenectomy OR pancreatic surgery). For the Scopus database, the following combination was used: TITLE-ABS-KEY (“shunt OR shunting OR shunts”) AND TITLE-ABS-KEY (“pancreatectomy OR pancreaticoduodenectomy OR pancreatic surgery”). The same keywords were inserted into the search manager fields of Cochrane Library Central.

The search was limited to studies in humans published in English; no restriction criteria were set for study type or design. For all the databases, the last search was run on 18 June 2024. The reference list of the articles retrieved was further checked to identify additional pertinent studies. Prisma statement guidelines for conducting and reporting systematic reviews were followed and the research protocol was registered at the International Prospective Register of Systematic Reviews (http://www.crd.york.ac.uk/PROSPERO) (accessed on 1 May 2024) with the following registration number: CRD42023464951.

### 2.2. Study Selection and Data Extraction

Each study retrieved, reporting at least one short- or long-term outcome, was included in the analysis, regardless of the age or sex of the patients. Exclusion criteria were: (a) lack of sufficient data or outcomes of interest; (b) redundant publications from the same institution; and (c) creation of vascular shunts during surgical procedures other than pancreatic surgery (e.g., transplantations). In the case of two or more articles reporting outcomes from the same institution, only the most recent study or the one with the highest quality was included in the analysis; the second article was included if reporting different outcomes. Cases of disagreement between the two researchers were solved via discussion until a consensus was reached or by consulting a third author (MGS). In this systematic review, we included only articles published after August 2006, when the first paper on CT-defined criteria of locally advanced PDAC was published [8], followed by the definition of AHPBA/SSO/SSAT published in 2009 [8,9], and criteria published by the International Study Group of Pancreatic Surgery (ISGPS) in 2014 [10].

### 2.3. Outcomes

The main outcomes evaluated were the overall complication rate and the shunt patency. Additional outcomes of interest were as follows: R0 resection rate, in-hospital stay (days), operative time (min), intraoperative blood loss (mL), and 90-day mortality.

### 2.4. Meta-Analysis

All references selected for retrieval from the databases were managed with the MS Excel data collection software platform by a senior biostatistician (FC). The data extracted from the selected studies and stored in the database were structured as evidence tables. Statistical analyses were conducted using the meta-package (R software, version 2023.03.1). The outcomes reported in Table 1 were analyzed to provide an overall estimate of the studies included in a single-arm meta-analysis of incidences and continuous outcomes. The outcomes of interest analyzed meta-analytically were the number of shunt patency, surgery complications, and subjects with R0 resection rate, as proportions while operative time (min), and intraoperative blood loss (mL) as mean values (Supplementary Material).

The R package “meta” was used to calculate the overall value within the studies included in the meta-analysis. In particular, the metaprop function was used for the calculation of the overall proportion for the above-described categorical variables and the metamean function for the estimates of the overall averages of the indicated continuous variables. Heterogeneity was assessed via Cochran’s Q statistic and quantified (*I*^2^). The *I*^2^ statistic and P value were used to analyze study heterogeneity. A *p*-value < 0.1 or *I*^2^ > 50 was considered as meaningful heterogeneity between studies. Random effects models were applied for all analyses. All analyses were performed with R software version 223.03.1 by a senior biostatistician (FC).

### 2.5. Risk of Bias Assessment

The Newcastle–Ottawa scale was used for the assessment of study quality and risk of bias for nonrandomized studies, including case-control and cohort studies. This scale uses a star rating system to evaluate study group selection, comparability of groups, and ascertainment of the outcome of interest; the higher the number of stars awarded to a study (maximum 9 stars), the lower the risk of bias.

## 3. Results

**Figure 4:**
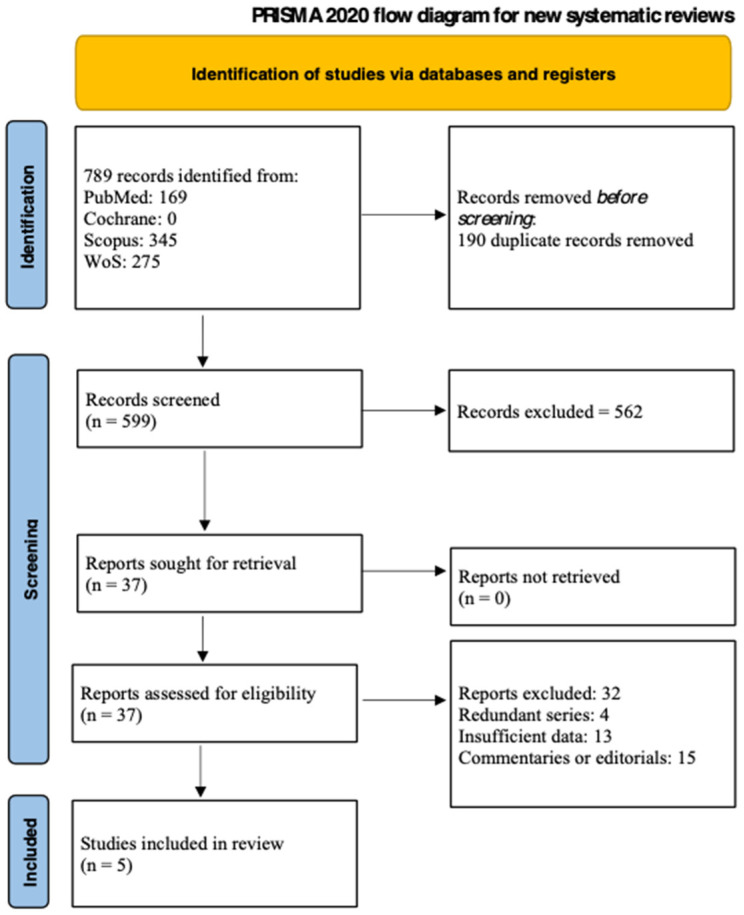
PRISMA 2020 flow diagram of the screening process.

The PRISMA flow diagram for the systematic review is shown in Figure 4. The database search identified 789 papers; after duplicate removal, 599 abstracts were screened. Of these, 561 were excluded because were deemed not relevant to the purpose of the study. Then, 38 articles were selected for full-text review, and of these, 33 were excluded for the following reasons: four papers were redundant series from the same institutions [15,16,17,18]; 13 were articles, abstracts, or posters with insufficient data [19,20,21,22,23,24,25,26,27,28,29,30,31]; and 15 were comments or editorials concerning surgical techniques [32,33,34,35,36,37,38,39,40,41,42,43,44,45,46]. Finally, five papers satisfied the selection criteria and were included in the systematic review [6,11,12,13,14]. Table 1, Table 2, Table 3 and Table 4 show the characteristics of these studies. Four were cohort studies [6,13,14,18] and one was a case-control study [11]; all the papers were monocentric studies with a retrospective design. From 2008 to 2019, a total of 145 patients had operative shunts during pancreatic resections.

Data regarding preoperative characteristics of patients were reported in all five studies and included ASA score [13,14,18], BMI [11,13], ECOG performance status, and Charlson Comorbidity Index [6] and preoperative CA19-9 [11,12,14]. PDAC was the main diagnosis in the five studies, accounting for 88% of the global cohort (128 patients), and neoadjuvant therapy was performed in 73% of patients with PDAC.

Pancreaticoduodenectomy was the most common surgical procedure (accounting for 83% of the entire cohort), followed by total pancreatectomy and extended pancreatoduodenectomy (seventeen and seven cases, respectively).

Distal splenorenal shunt (DSRS) was the most performed shunt (109 patients, 76% of cohort), followed by MPS (15 cases, 10%) and MCS (16 cases, 11%); two cases of combined DSRS-MCS shunt (2%) and 1 case of splenic-adrenal shunt (1%) were also reported, along with a rare case of combined shunt between left gastric vein and inferior vena cava in combination with a shunt between inferior mesenteric vein and inferior vena cava, described by Oehme and colleagues [14].

In 56 cases there was an arterial involvement requiring synchronous arterial resection. Only two articles reported the time of clamping necessary to achieve shunt creation. Bachellier and colleagues [12] reported 25 ± 2 min (range, 18–27) for TMPS and Al-Saeedi and colleagues [13] reported 5–15 min for DRSR creation.

Moreover, each study reported the surgical procedure adopted to reconstitute SMP confluence: Addeo [11], Al-Saeedi [13], and Bachellier [12] described primary end-to-end PV-SMV anastomosis, Chavez [6] reported 13 cases of LIJV interposition graft, and Oehme [14] reported both primary anastomosis and LIJV interposition graft.

Three out of five studies described the anticoagulation management: Al-Saeedi [13] reported the standard use of low-molecular-weight heparin (LMWH) for thrombo-prophilaxis after surgery until discharge and no antiplatelets agent use; Bachellier [12] reported no systemic heparinization during and after surgery; Chavez [6] reported use of intraoperative systemic heparinization, aspirin per rectum in the recovery room, postoperative subcutaneous unfractionated heparin, and aspirin at discharge in case of venous graft.

Three out of five papers managed SV stump through a DSRS; Bachellier [12] reported nine end-to-end SV-IMV anastomosis, three end-to-end SV-left renal vein anastomosis, three cases of no anastomosis due to distal insertion of IMV into SV; and Oehme [14] reported one case of anastomosis between SV and IVC.

Mean operative time ranged from 430 min to 659 min. Chavez [6] reported the median operative time of 540 min (range 390–780); Oehme [14] reported a median operative time of 566 min (range 456–671). The pooled estimate for operative time, with random effects model was 564.35 (95% CI 500.01 to 628.68) (Supplementary material). Four research papers reported blood loss: 950 mL (median, 200–5.000 mL) for Chavez manuscript [6], 850 mL (mean, 420–4.300 mL) for Bachellier and colleagues [6,12], 1200 mL ± 600 mL (mean) according to Al-Saeedi [13], and 1800 mL (median, 525–3075) for Oehme [14]. The pooled estimate for blood loss was 1227.65 (95% CI 804.18 to 1651.11) for the model adopted (Supplementary Material).

Complications were described in all five studies: overall, 44/145 patients (30% of the entire cohort) reported postoperative complications. Addeo [11] reported mean overall morbidity of 30% (23/78 patients) whereas Bachellier [12] reported mean overall morbidity of 47% (7/15 patients); Al-Saeedi [13] reported two Clavien–Dindo 1–2 complications and three Clavien–Dindo 3–4 complications (5/10 patients, 50%); Chavez [6] reported major complications only (Clavien > 3), accounting for four cases in 31 patients. Similarly, Oehme [14] reported five cases of major complications (Clavien > 3). The pooled estimate for adverse events with random effects models was 32% (95% CI 21–47%) (Supplementary Material).

Long-term patency of definitive shunts was reported in four studies with an overall rate of 83% (110/120 patients): Chavez [6] reported the occlusion of one DRSD among 20 definitive shunts and the patient was asymptomatic; Al-Saeedi [13] reported a long-term patency rate of DSRS of 60% (four occlusions of DRSS) and similarly, none of the patients developed signs and complications of LPH; Addeo [11] reported two occlusions of DRSR with a consequent increase in splenic volume. Oehme [14] reported the long-term patency of two DSRS, one splenocaval shunt, and one combined shunt, taking into account that three patients out of eleven were lost during follow-up. The pooled estimate for the shunt patency, with random effects models, was 87% (95% CI 59 to 97%) (Supplementary Material).

Al-Saeedi [13] reported a mean length of hospital stay (LOS) of 27.2 +/- 23.4; Chavez [6] reported a median LOS of 11 days (7–35). Mortality was reported in the five papers included and accounted for three patients for the entire cohort. The mean R0 resection rate was 62% of the entire cohort, considering that PDAC was the main but not the unique preoperative diagnosis. The pooled estimate for the R0 resection rate, with random effects models, was 76% (95% CI 43–93%) (Supplementary Material). Median overall survival was reported in four studies: Addeo [11] reported a median OS of 22 (14–27) months, Chavez [6] of 31 (6.4–101) months for patients with PDAC, Bachellier [12] of 17 months, and Oehme [14] reported a median postoperative survival of 12 months.

### Risk of Bias Assessment

**Figure 5:**
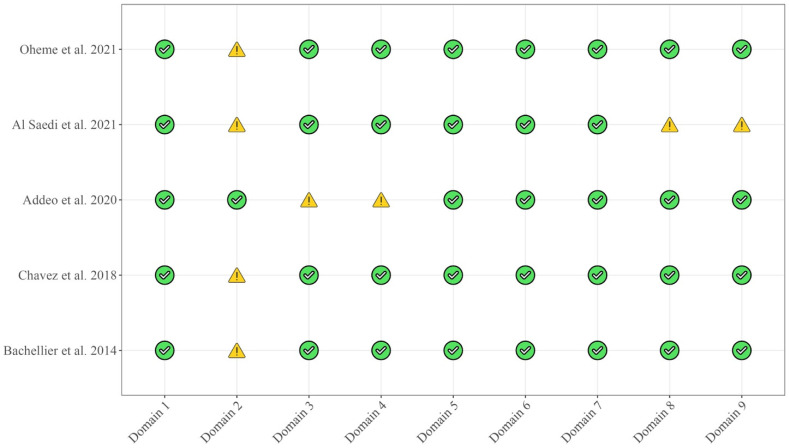
Risk of bias assessment according to Newcastle and Ottawa scale [6,11,12,13,14].

**Figure 6:**
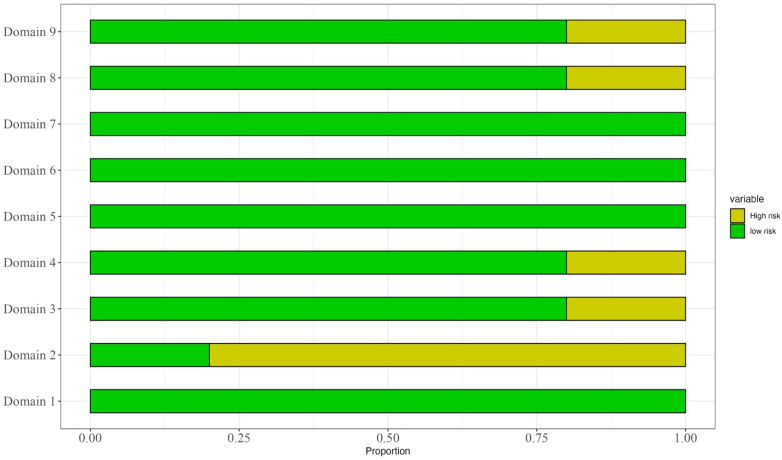
Risk of bias of selected studies across domains.

The papers were evaluated according to the Newcastle and Ottawa scale, as shown in Figure 5 and Figure 6. The quality of the studies was moderate to good, and the only concerns were about domains 2, 3, 4, 8, and 9.

## 4. Discussion

The use of operative shunts during pancreatic resection was first described in the late 20th century by Asiatic authors reporting pump-assisted venous bypass [47,48,49,50]. Interest in such procedures rapidly waned, due to difficulties in the availability of equipment and reproducibility of technique but has come back thanks to high response rates of PDAC to neoadjuvant therapy and consequent potential resection [51].

Vascular resection for borderline and locally advanced PDAC has gradually gained diffusion, becoming a standardized procedure in high-volume centers with acceptable morbidity and mortality [10,52]. Results from a recent review [53] have shown that pancreatectomy combined with vascular resection required longer operative time and increased perioperative blood loss compared to standard PD, but postoperative complications were similar to those of PD without vascular resection and interestingly, patients who had vascular resection experienced less pancreatic fistula. This latter outcome is in line with further evidence in the literature [54] and explains the surprisingly lower fistula rate reported in patients who underwent PD with vascular resections as a consequence of more fibrotic pancreatic texture in this subgroup of patients. Wang and colleagues [53] reported a mean operative time of 491 min (ranging from 342 to 667), average blood loss of 929 mL (ranging from 343 to 1686), and overall postoperative complications of 37% (range 20.7–55.6%). Our analysis refers to a very small fraction of patients who underwent pancreatic surgery with vascular resection and additional shunt creation, yet outcomes seem to be comparable. Regarding surgery duration, clamping time for shunt creation is negligible compared to the average operative time of the surgical procedure [12,13]. Moreover, none of the articles reported either intraoperative or postoperative procedure-related complications (e.g., bleeding, renal insufficiency, renal vein thrombosis, liver insufficiency), except for Chavez [6] who reported a case of mild encephalopathy after delayed postoperative MCS. Yet, two out of four papers included [11,12] did not report postoperative morbidity according to the Clavien–Dindo classification, making it difficult to make comparisons and draft conclusions about actual shunt-related morbidity.

The definition of left-sided portal hypertension (LPH) was given by Evans, referring to the syndrome characterized by bleeding of gastric and esophageal varices in patients with patent portal vein (PV) and normal hepatic function, due to isolated SV obstruction by various underlying etiologies (cystic lesions, neoplasms, pancreatitis, surgical ligation etc.) [55]. The mechanisms underlying LPH after pancreatic surgery with SV ligation are still under investigation, since this issue has been considered negligible for decades. Strasberg’s group [56,57] has made a meticulous mapping via postoperative CT scan of collateral veins which developed after PD with SV ligation, identifying two routes of alternative drainage of spleen and stomach: the superior route via the left gastric vein (LGV) and the inferior route via either the right colonic marginal vein arcade (SRCV arc) or the middle colic vein (MCV), draining into the superior mesenteric vein.

The abovementioned veins are [37,44] also identified as “critical veins” [37]. When decompression of the spleen and stomach is ensured by collaterals, such as left gastric vein (LGV), middle colic vein (MCV), or inferior mesenteric vein (IMV), the splenic vein may not be reimplanted [44,56]. However, these veins are often divided for oncological purposes or may not provide satisfactory decompression if preserved because they may not remain patent or functioning, with gradual development of collateral varices and consequent onset of left-sided portal hypertension.

The low incidence of LPH can be biased by several factors, first of all short-term follow-up and dismal survival of patients with PDAC, who may die before experiencing this long-term complication. Secondly, studies have heterogeneous definitions of LPH and its manifestation, in terms of the method used for spleen volume calculation, time of postoperative evaluation or platelets (PLT) count cut-off defining thrombocytopenia (PLT count < 150/nL for Al-Saeedi vs. PLT count < 100.000/mm^3^ for Addeo). Thirdly, the technique used for PD with vascular resection/reconstruction may differ among studies and influence the choice of whether or not to use a shunt, the type of shunt, and the venous reconstruction adopted. Extensive dissection of the retroperitoneum with the division of small collaterals, as described by the Strasbourg group with the Cattle–Braasch maneuver [11,12], may reduce alternative routes of drainage of the spleen and stomach. Similarly, the low incidence of LPH can also be explained by the fact that gastric congestion can be treated intraoperatively with partial gastrectomy which mitigates LPH [58]. Included studies may be biased by the use of partial gastrectomy (e.g., five out of ten patients in the Heidelberg cohort) [13] and pancreaticogastrostomy [11,12] which benefited more from DSRS, ensuring gastric drainage and may explain the low frequency of LPH and improved outcomes of DSRS population compared to those with SV ligation.

The authors believe that, as the survival of patients requiring pancreatectomy with vascular resection is improving thanks to the development of chemotherapy and surgical techniques, LPH is expected to become an increasingly frequent complication after PD. Therefore, reasonable criteria for reimplantation of SV must be established on solid clinical evidence and not rely on the surgeon’s experience and intraoperative discretion. An attempt was made by Tanaka and colleagues [44] who stratified the risk of postoperative LPH according to the number and type of preserved critical veins. In this cohort, patients in whom none of these three critical veins were preserved and no SV reconstruction was performed, all patients (29/29) developed LPH; in patients with only one critical vein preserved and no reconstruction, 24% of them (12/51) developed LPH. In contrast, no LPH developed when one critical vein was preserved and SV was reimplanted. Finally, no patients with preservation of two or three critical veins (*n* = 8) developed LPH. Therefore, it seems that not only the number of critical veins preserved, but also their haemodynamic evaluation (e.g., caliber, flow etc.,) may affect splenic drainage and LPH development.

The decision whether to reimplant SV or not, the prediction of the risk of developing LPH, the identification of a spontaneous splenorenal shunt which is often undetectable on preoperative imaging, and the selection of the “ideal patient” better suited to such aggressive procedures are some of the new dilemmas of advanced pancreatic surgery. The included studies reported experience in high-volume referral centers, where surgery is performed by surgeons with extensive expertise in HPB, vascular, and transplant surgery, together with a multidisciplinary team of dedicated oncologists, radiologists, and anesthesiologists. Patients are well selected after a long course of neoadjuvant treatment (76% of patients affected by PDAC in the entire cohort), which helps to screen those with favorable tumor biology; they usually have good performance status, a low burden of comorbidities, and adequate familiar and social context to successfully recover from major surgery [6].

However, most of the decision process is based upon variables which are dependent on personal discretion and judgment (e.g., intraoperative pancreatic texture, radiological assessment of vascular infiltration) or can be assessed at the time of surgery (e.g., estimated blood loss). Artificial intelligence has the potential to shape clinical decisions, improve outcomes and minimize the risk of errors [59]. Radiomics is one of the most promising fields for the application of artificial intelligence [59,60] and consists of an image analysis (e.g., ultrasounds images, CT scan, MRI) mostly based on machine learning (ML) algorithms, that aims to obtain an objective characterization of biological tissues by converting images into extractable data. Despite the low quality of most radiomics studies published so far, preliminary results are exciting. ML algorithms, for example, can outperform human interpretation of images and recognize features ``hidden to human eyes”, such as the difference between perineural invasion and true arterial infiltration, which obviously affects surgical strategy and R0 resection [59,60,61]. Moreover, recent studies aimed to correlate radiomics features with tumor response after neoadjuvant chemotherapy or with the risk of recurrence after surgical resection of PDAC, in order to avoid futile surgery [59,60,61,62]. The studies included in this systematic review [6,12,13] asserted that preoperative protocols provide CT-scan with 3D reconstruction besides standard coronal and sagittal reformations, stressing that shunt use, far from being improvised, is preoperatively planned upon exhaustive study of relationship between the tumor, the vascular structures, and the pancreas itself. Visualization via 3D and artificial intelligence [63] have an increasing application also during surgery, where navigation techniques can facilitate SMA identification and venous resection-reconstruction.

Surgical workflow can also be enhanced by indocyanine green (ICG) administration. In the first-published consensus on fluorescence-guided surgery (FGS) for PDAC [63,64], all experts agreed that FGS is safe with few drawbacks during PDAC surgery. The main limitation consists of the lack of selectiveness of ICG for PDAC, therefore it should not yet be used routinely for tumor identification. Besides this limitation, ICG can be used to assess vitality of surrounding organs (e.g., colon, stomach, spleen), improve visualization of vascular structures, or ease the identification of hepatic micrometastases. Research is necessary to determine the optimum dose, concentration, and timing of specific near-infrared tracers for each specific purpose.

Some limitations should be accounted for such as the low number of patients involved, the short-term follow-up, and the long time of enrollment comprising eleven years from 2008 to 2019. Validation of these encouraging results on a large scale in a longitudinal setting is highly desirable.

## 5. Conclusions

An increasing number of patients with borderline resectable or locally advanced PDAC are becoming amenable to resection, and shunt creation in expert hands may facilitate vascular resection with clear margins, which is the only chance for long-term survival.

## Figures and Tables

**Table 1:** Intraoperative and postoperative outcomes across selected studies (*n* = 5).

Author, Year	Operative Time (min)	Blood Loss (mL)	Complications	Shunt Patency	LOS Days	R0	Mortality
Addeo et al. [11]	600 +/- 128	NR	23/78	76/78	NR	36/78	1
Chavez et al. [6]	Median 540 (390–780)	Median 950 mL (200–5.000)	4/31 _*_	19/20	Median 11 days (7–35)	30/31	0
Bachellier et al. [12]	659 +/- 118	850 +/- 1200 mL (range 420–4300)	7/15 _§_	NA	NR	12/15	0
Al-Saeedi et al. [13]	430.7 ± 146.2	1200 ± 600	5/10 _#_	6/10	27.2 +/- 23.4	3/10	1
Oehme et al. [14]	Median 566 (459–671)	Median 1800 mL (525–3075)	5/11 °	4/7	NR	10/11	1

Abbreviations: NR: not reported; NA: not applicable; LOS: length of the hospital stay. * Chavez and colleagues reported major postoperative complication (Clavien–Dindo > 3): 2 fluid collection drainage, 1 cardiopulmonary arrest, 1 reoperation due to coagulopathy. § Bachellier and colleagues reported all type of complications regardless of their treatments and Clavien–Dindo classification: 2 pleural effusion, 2 sepsis, 1 liver necrosis of segment 5, 1 chylous ascites, 1 splenic thrombosis, # Al-Saeedi and colleagues reported all type of complications: 2 cases of Clavien 1–2, 3 cases of Clavien > 3, ° Oehme and colleagues reported major postoperative complication (Clavien–Dindo > 3): 2 relaparotomy for hemorrhage or leak of gastrojejunostomy, 2 fluid collection drainage, 1 sepsis.

**Table 2:** Neoadjuvant treatments across selected studies (*n* = 5).

Author, Year	Neoadjuvant Therapy	
	N. Patients	Type of Neoadjuvant Therapy
Addeo et al. [11]	45/78	Chemotherapy
Chavez et al. [6]	29/31	26 PDAC chemotherapy+chemoradiation, 1 PDAC chemotherapy. 1 PNET chemotherapy, 1 PNET chemo+chemoradiotherapy
Bachellier et al. [12]	15/15	Chemotherapy (8 FOLFIRINOX + 7 GEMOX)
Al-Saeedi et al. [13]	3/10	Chemotherapy/radiotherapy
Oehme et al. [14]	4/11	Chemotherapy (3 FOLFIRINOX, 1 gemcitabine)

Abbreviations: PDAC: pancreatic ductal adenocarcinoma; PNET: pancreatic neuroendocrine tumor.

**Table 3:** Shunt creation and technical aspects across selected studies (*n* = 5).

Author, Year	Associated Arterial Resection	Surgery	Shunt	Type of Shunt	Time of Clamping	Duration of Shunt	Anticoagulation	Reconstruction
Addeo et al. [11]	20/78 SMA, 14/78 HA	78 PD	78 DSRS	end-to-side SV-LRV anastomosis	NR	Definitive	NR	SMV-PV direct anastomosis
Chavez et al. [6]	2 CHA	PD 18, TP 6, EXTENDED PD 7	16 DSRS, 12 MCS, 2 COMBINED, 1 SAS	14 LIJV	NR	Temporary, Definitive	Heparin and aspirin	Primary end-to-end SMV-PV; SMV-PV with internal jugular vein interposition graft, SMV-IJV-IVC 3 patients who had permanent MCS
Bachellier et al. [12]	7 SMA, 2 CHA, 1 CHA + LHA, 1 CT	12 PD, 3 TP	15 TMPS	Gore-Tex	25 ± 2 min (range, 18–27)	Temporary	No systemic heparinization during and after	End-to-end PV/SMV anastomosis
Al-Saeedi et al. [13]	4/10	6 PD, 4 TP spleen preserving	10 DSRS	end-to-side SV-LRV anastomosis	5–15 min	Definitive	Thrombosis prophylaxis with low molecular heparin weight following surgery until discharge, No use of antiplatelet agent	SMV-PV end-to-end 5–0 or 6–0 monofilament nonabsorbable running suture
Oehme et al. [14]	5 CHA, 1 RHA	7 PD, 4 TP	5 DSRS, 4 MCS, 1 SCS, 1 COMBINED *	DSRS (end-to-side SV-LRV anastomosis), MCS (3LIJV, 1 bovine patch)	NR	Temporary, Definitive	NR	Primary end-to-end SMV-PV; SMV-PV with internal jugular vein interposition graft

Abbreviations: SMA: superior mesenteric artery; HA: hepatic artery; CHA: common hepatic artery; LHA: left hepatic artery; RHA: right hepatic artery; CT: celiac trunk; PD: pancreatoduodenectomy; TP: total pancreatectomy; DSRS: distal splenorenal shunt; MCS: mesocaval shunt; SAS: splenoadrenal shunt; SCS: splenocaval shunt; TMPS: temporary mesoportal shunt; SV: splenic vein; LRV: left renal vein; LIJV: left internal jugular vein; NR: nor reported. * Oehme and colleagues reported one anecdotal case of combined shunt in 1 patient consisting in a shunt between left gastric vein and inferior vena cava in combination with a shunt between inferior mesenteric vein and inferior vena cava.

**Table 4:** Preoperative characteristics of patients across selected studies (*n* = 5).

Author, Year	Study Design	Study Location	Time of Enrollment	N	F	M	Age	Diagnosis
Addeo et al. [11]	Case-control	France	2012–2018	78 _*_	NR	NR	63 +/- 8.1	68/78 PDAC
Chavez et al. [6]	Cohort	USA	2009–2018	31 _§_	16 _#_	18	Median 61 (21–80) _#_	27 PDAC, 3 PNET, 1 SPP _**_
Bachellier et al. [12]	Cohort	France	2008–2012	15	6	9	65 +/- 18 (46–83)	Locally advanced PDAC
Al-Saeedi et al. [13]	Cohort	Germany	2017–2019	10	5	5	64 +/- 8	9 PDAC, 1 PNET
Oehme et al. [14]	Cohort	Germany	2012–2017	11	8	3	Median 65.1 (57–73.5)	9 PDAC, 1 dedifferentiated, 1 pleomorphic

Abbreviations: NR: not reported; PDAC: pancreatic ductal adenocarcinoma; PNET: pancreatic neuroendocrine tumor; SPP: solid pseudopapillary tumor. * Addeo and colleagues reported data of 114 patients: 78 of them received distal splenorenal shunt, 36 underwent splenic vein ligation without reimplantation. § Chavez reported data of 34 patients: 31 of them received shunt at the time of surgery, 3 were submitted to shunt creation after index pancreatectomy # Data regarding entire cohort of 34 patients ** Data regarding 31 patients who received shunt creation at the time of pancreatectomy.

## Data Availability

All data supporting the findings of this study are available from the corresponding authors (A.L.) upon reasonable request.

## Supplementary Materials

The following supporting information can be downloaded at: https://www.mdpi.com/article/10.3390/cancers16132361/s1.

## Abbreviations

Pancreatic adenocarcinoma (PDAC); splenic-mesenteric-portal confluence (SMP confluence); portal hypertension (PH); portal vein (PV); mesocaval shunt (MCS); inferior vena cava (IVC); superior mesenteric vein (SMV); left internal jugular vein (LIJV); mesoportal shunt (MPS); superior mesenteric artery (SMA); distal spleno-renal shunt (DSRS); splenic vein (SV); left renal vein (LRV); splenocaval shunt (SCS); left-sided portal hypertension (LPH); Americas Hepato-Pancreato-Biliary Association (AHPBA); Society of Surgical Oncology (SSO); Society for Surgery of the Alimentary Tract (SSAT); International Study Group of Pancreatic Surgery (ISGPS); hepatic artery (HA); common hepatic artery (CHA); left hepatic artery (LHA); right hepatic artery (RHA); celiac trunk (CT); pancreaticoduodenectomy (PD); left gastric vein (LGV); middle colic vein (MCV); inferior mesenteric vein (IMV); right colonic marginal vein arcade (SRCV arc); platet (PLT); machine learning (ML); indocyanin green (ICG); fluorescence-guided surgery (FGS).
