# Supplementary figures and images for: Use of Vascular Shunt at the Time of Pancreatectomy with Venous Resection: A Systematic Review
Source: Cancers (Basel). 2024 Jun 27;16(13):2361. doi: 10.3390/cancers16132361 (PMC11240683; doi:10.3390/cancers16132361)

## Operative Time

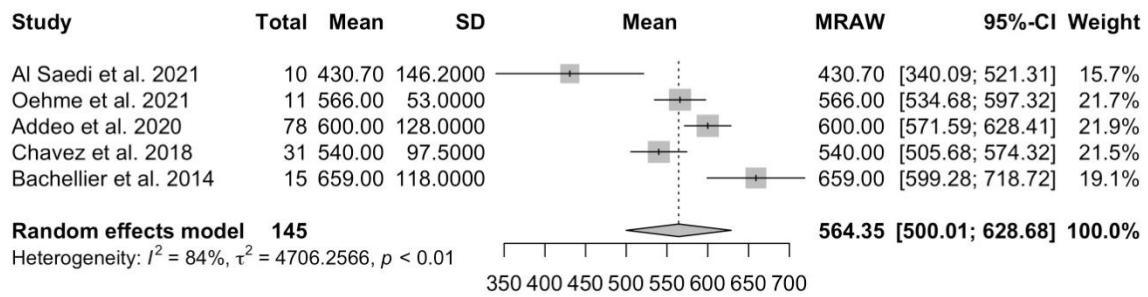

## Blood loss

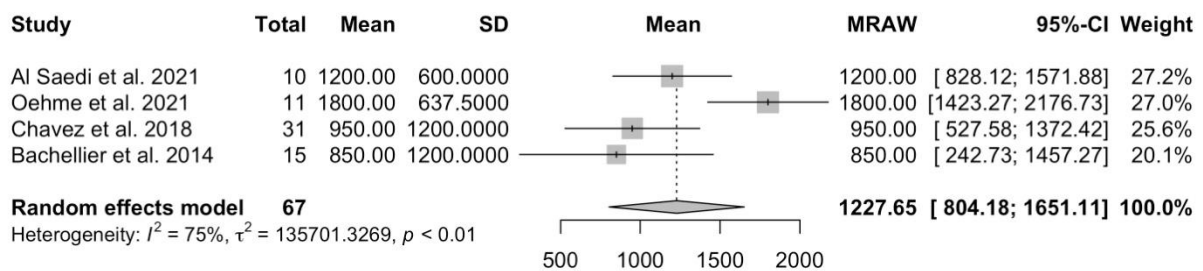

## Shunt Patency

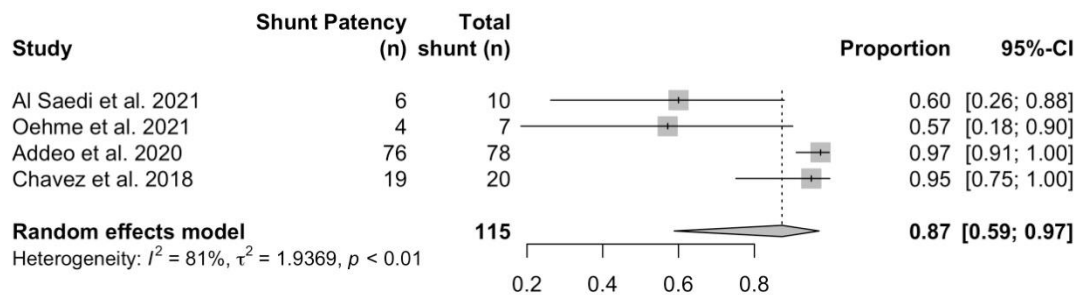

## Complications

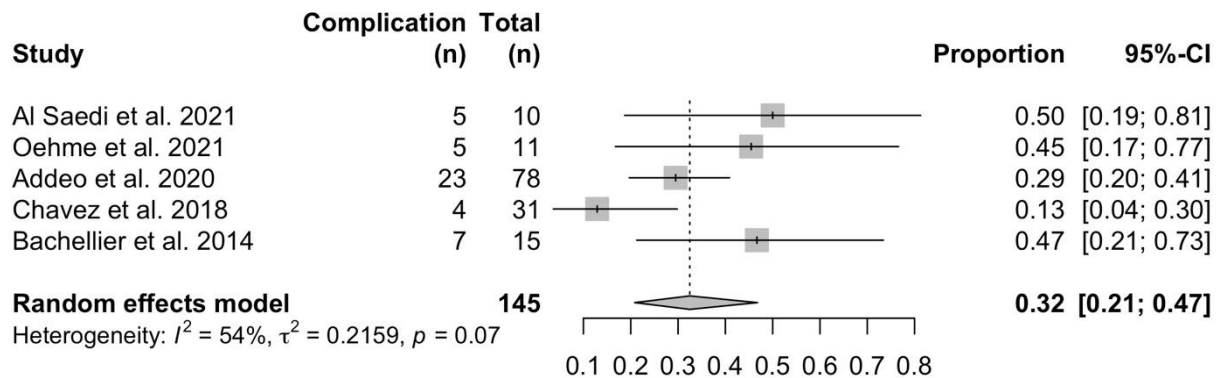

R0

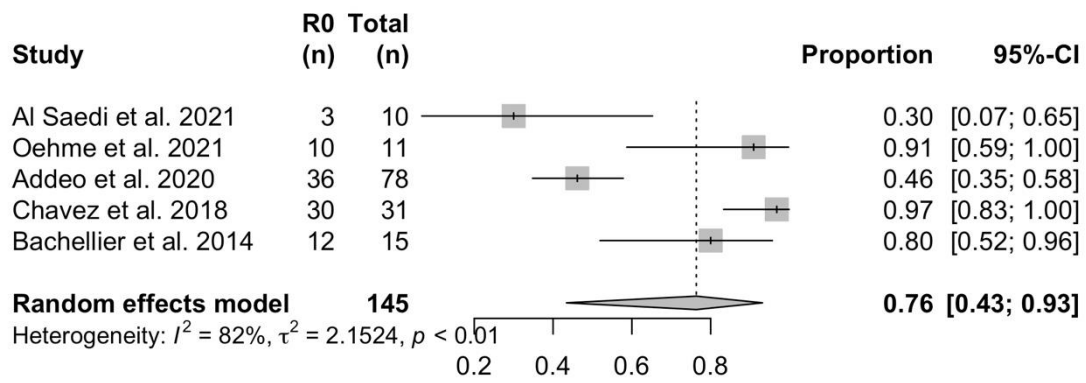

Supplement: Supplementary file 1 [file cancers-16-02361-s001.zip › cancers-3050693-supplementary.pdf]
